# Artificial Intelligence in Cardiac MRI: Is Clinical Adoption Forthcoming?

**DOI:** 10.3389/fcvm.2021.818765

**Published:** 2022-01-10

**Authors:** Anastasia Fotaki, Esther Puyol-Antón, Amedeo Chiribiri, René Botnar, Kuberan Pushparajah, Claudia Prieto

**Affiliations:** ^1^Department of Biomedical Engineering, School of Biomedical Engineering and Imaging Sciences, King's College London, London, United Kingdom; ^2^Guy's and St. Thomas' NHS Foundation Trust, London, United Kingdom; ^3^Escuela de Ingeniería, Pontificia Universidad Católica de Chile, Santiago, Chile

**Keywords:** cardiac MRI, artificial intelligence, clinical integration, neural network, machine learning

## Abstract

Artificial intelligence (AI) refers to the area of knowledge that develops computerised models to perform tasks that typically require human intelligence. These algorithms are programmed to learn and identify patterns from “training data,” that can be subsequently applied to new datasets, without being explicitly programmed to do so. AI is revolutionising the field of medical imaging and in particular of Cardiovascular Magnetic Resonance (CMR) by providing deep learning solutions for image acquisition, reconstruction and analysis, ultimately supporting the clinical decision making. Numerous methods have been developed over recent years to enhance and expedite CMR data acquisition, image reconstruction, post-processing and analysis; along with the development of promising AI-based biomarkers for a wide spectrum of cardiac conditions. The exponential rise in the availability and complexity of CMR data has fostered the development of different AI models. Integration in clinical routine in a meaningful way remains a challenge. Currently, innovations in this field are still mostly presented in proof-of-concept studies with emphasis on the engineering solutions; often recruiting small patient cohorts or relying on standardised databases such as Multi-ethnic Study on atherosclerosis (MESA), UK Biobank and others. The wider incorporation of clinically valid endpoints such as symptoms, survival, need and response to treatment remains to be seen. This review briefly summarises the current principles of AI employed in CMR and explores the relevant prospective observational studies in cardiology patient cohorts. It provides an overview of clinical studies employing undersampled reconstruction techniques to speed up the scan encompassing cine imaging, whole-heart imaging, multi-parametric mapping and magnetic resonance fingerprinting along with the clinical utility of AI applications in image post-processing, and analysis. Specific focus is given to studies that have incorporated CMR-derived prediction models for prognostication in cardiac disease. It also discusses current limitations and proposes potential developments to enable multi-disciplinary collaboration for improved evidence-based medicine. AI is an extremely promising field and the timely integration of clinician's input in the ingenious technical investigator's paradigm holds promise for a bright future in the medical field.

## Introduction

### Artificial Intelligence

(AI) is an academic discipline founded in the early 1950's and is considered as any method that allows computers to accomplish functions, that require human intelligence. AI introduces speed in performing tedious and time-consuming tasks, precision in tasks requiring analysis and can draw sophisticated interconnections/ deep interpretation of digital data. It is already widely adopted in various scientific fields including space craftmanship, navigation, meteorology and every-day tasks including social media, banking, digital voice assistants (1–3). The clinical uptake of the advances made by computer scientists and engineers has been progressive but slow.

Cardiovascular Magnetic Resonance imaging (CMR) is already an established tool for routine clinical decision-making including diagnosis, follow-up, pre-procedural planning and real-time procedures. It is ideally suited for various AI techniques due to the digitalisation of the MRI signal and the diversity in the contrast and parametric information that can be obtained from the images.

This review article explores the basic AI concepts that are currently adopted in CMR along with relevant clinical applications. We have only included studies that are prospectively designed and applied. The aim is to familiarise clinicians with the basics in AI, demonstrate the feasibility of relevant applications and discuss current shortcomings that could be addressed in future work.

### AI Basics

Machine learning (ML) is a subcategory of AI that teaches computers to do what humans and animals naturally do: learn from experience. ML uses algorithms to find patterns and make extrapolations from large amounts of data. The algorithms adaptively enhance their performance as the amount of datasets for learning expands. In the workflow of ML, feature extraction is the first step, and this is followed by the development of the model. The accuracy of the ML model is highly dependent on the features extracted.

**Figure 1:**
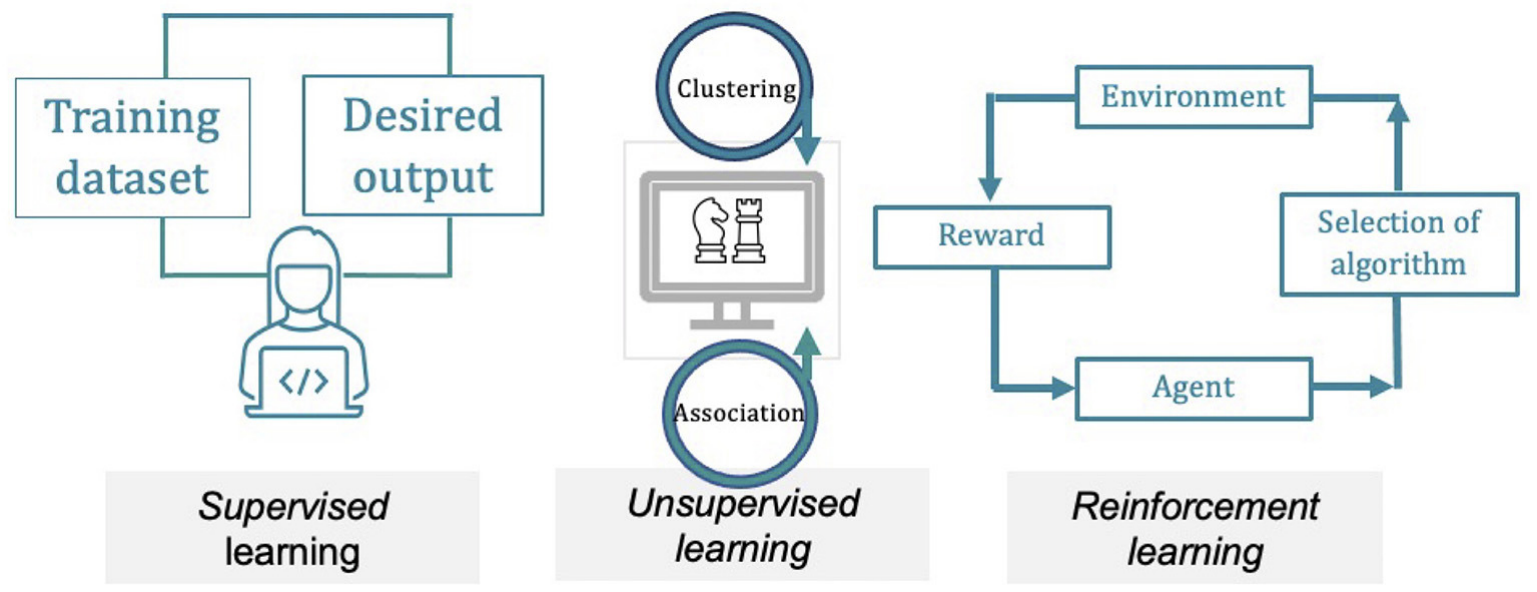
Simplified graph describing the three principal machine-learning methods. Supervised learning utilises hand-labelled datasets to design algorithms that predict future events, classify data into defined categories or distinguish the most relevant variables to the output. The predictive model learns through data training and improves over time. In unsupervised learning the software accomplishes the processing of raw data, finding hidden structures in datasets, without prior annotation, identifying meaningful relationships and clusters within the data. Reinforcement learning is a reward-based learning. Its foundation lies in the interactions with an environment, in which positive and negative feedback (reinforcements) contribute to the optimisation of the model.

ML is further divided into supervised learning, unsupervised learning and reinforcement learning. The differentiation lies on the extent and type of supervision that is provided to the algorithms during training. Supervised learning uses datasets, annotated by a knowledgeable supervisor, to create models that predict or categorise future events or identify the most appropriate patterns to the outcome (4). The progress of the predictive model is dependent on the diversity of the data used in training along with the underlying algorithm. In unsupervised learning the computer programme is able to identify hidden structures in collections of databases, without previous labelling. The software can potentially determine novel relationships and clusters inside the data. Reinforcement learning constitutes a computational path to learn through interactions with the environment. It is a reward-based learning model, where positive and negative feedback contribute to the creation of effective predictive models (see Figure 1 for a schematic approach to the different types of ML categories).

Deep learning (DL) is a subset of ML that applies neural networks with hidden layers to correlate between the given input and the correct output, so that feature extraction and model development are performed simultaneously. DL algorithms are inspired from the network and the connections of the biological neurons in the brain that enable cognitive tasks. The nodes in a neural network mimic the neuronal function, i.e., they receive input signals, that can be excitatory or inhibitory, causing them to fire or withhold an output respectively. In mathematical terms, a neuron in the AI field is a placeholder for a numerical expression, which creates an output by applying the function on the given inputs. The data are progressively processed and fine-tuned through this hierarchy to extract high level features from simplified data. The predictive properties of the algorithm are learnt through a sequence of iterations.

**Figure 2:**
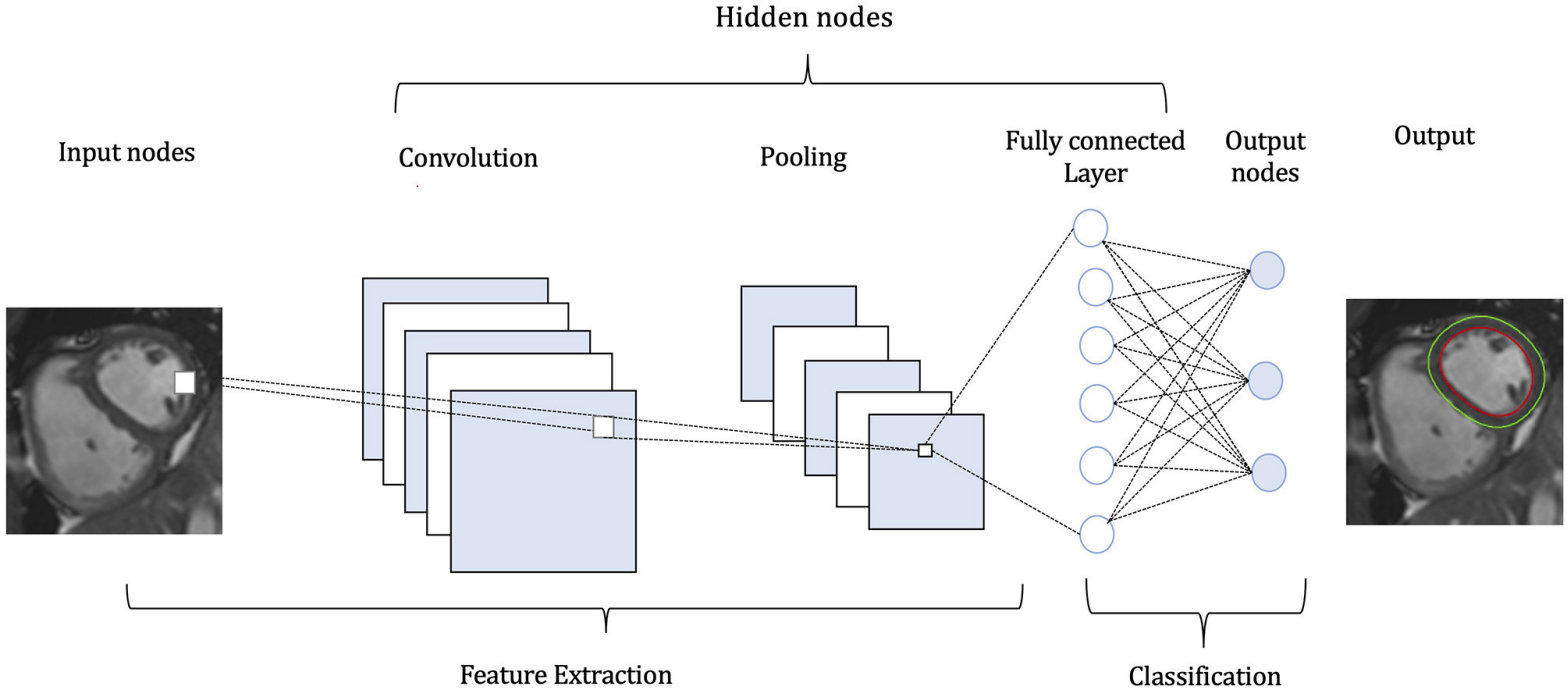
Pipeline of a convolutional neural network (CNN). A CMR image functions as input to the CNN. The CNN identifies and classifies the various attributes (features) of the image for analysis in a procedure named Feature Extraction, including a stack of convolutions and pooling operations. In the convolution operation different-level features, such as edges, colour, gradient orientation are extracted from the input image. The pooling layer reduces the dimensionality of the convolved features, in order to decrease the computational requirements. The nodes in the fully-connected layer are connected directly to all nodes in the previous layer. This layer compiles the data extracted by previous layers and applies various filters to form the final output.

Convolutional neural network (CNN) is a popular subgroup of DL networks, widely applied in CMR, as it is designed to work with imaging data (Figure 2). Several characteristics have made this technique more adaptive compared to conventional ML methods. While in ML methods the learned weights are manually engineered, after sufficient training, CNN can extract features automatically (i.e., learn filters), enabling the enhanced feature extraction to be a section of the classification learning process. CNN learns multiple features in parallel for a given input. Therefore, the data-mining needed in a CNN is lower, in contrast to other algorithms and it requires minimal human intervention (5). The architecture of CNN consists of three layers: (1) convolutional (feature extraction), (2) pooling (reduction in the number of input variables), and (3) fully-connected layer (connects neurons between layers). The convolutional layer, being the first layer, applies the mathematical operation of convolution, that is several filters to the input variable in order to recognise a large number of relevant features. The pooling layer minimises the size of the convolved feature map, thereby reducing the overall computational demands and costs of the network. The fully-connected layer connects the neurons between different layers. Based on the type of the data and the required accuracy, the network is optimised by iterating the convolution-pooling series numerous times. In any DL method, evaluating the loss function is a significant process, in order to warrant that the algorithm will model the data in the expected way. From a simplified viewpoint, the loss function can be formulated as a function which determines the relation between two variables, namely the deviation of the predicted output from the ground truth output. The training of the convolutional neural networks comprises multiple iterations (known as epochs), which compare the performance of the training set against the validation one, diminishing the loss function. One epoch means that a new input sample from the training dataset will be assigned to the network, thus the weights of each convolutional layer will be optimised (6). Learning curves, which depict loss vs. epochs and accuracy vs. epochs, are utilised to optimally train the network.

### Present Clinical Motivation

CMR offers comprehensive assessment of cardiovascular disease and is a rapidly expanding imaging modality. A recent study showed a 10-year increase of 573% in the number of scans performed in UK (7). This rise comes with an exponential increase in the resources required to support this, including availability and time of experts for image acquisition, post-processing and reporting, along with scan-time cost. Novel developments in CMR, including high resolution, contrast- free coronary artery and congenital heart disease (CHD) imaging, quantitative multi-parametric and perfusion MRI and MRI-derived biomarkers necessitate a cost-effective and time-efficient strategy for their successful integration in clinical routine (8). AI can have a significant role in this, in view of its potential to accelerate MRI scanning, image post-processing and reporting, introduce novel biomarkers and incorporate those in decision-making and prognostication models. Acceleration in image acquisition can have additional benefits for patients with claustrophobia, anxiety and inability to follow breath-holding commands.

Furthermore, recent data illustrate disparities with regards to the access to CMR services around the globe. Scan and post-processing acceleration along with automated analysis through AI can facilitate wider availability of sustainable, faster and cheaper CMR, resulting in improvement in patient care in less privileged areas (9).

### Clinical Applications

ML algorithms have been optimised and introduced in all aspects of the imaging workflow and implemented prospectively in diverse patient cohorts (10). Extensive applications have been investigated in undersampled image-acquisition, automated analysis and post-processing and development of predictive models.

### Time-Efficiency

#### Acquisition and Reconstruction

**Figure 3:**
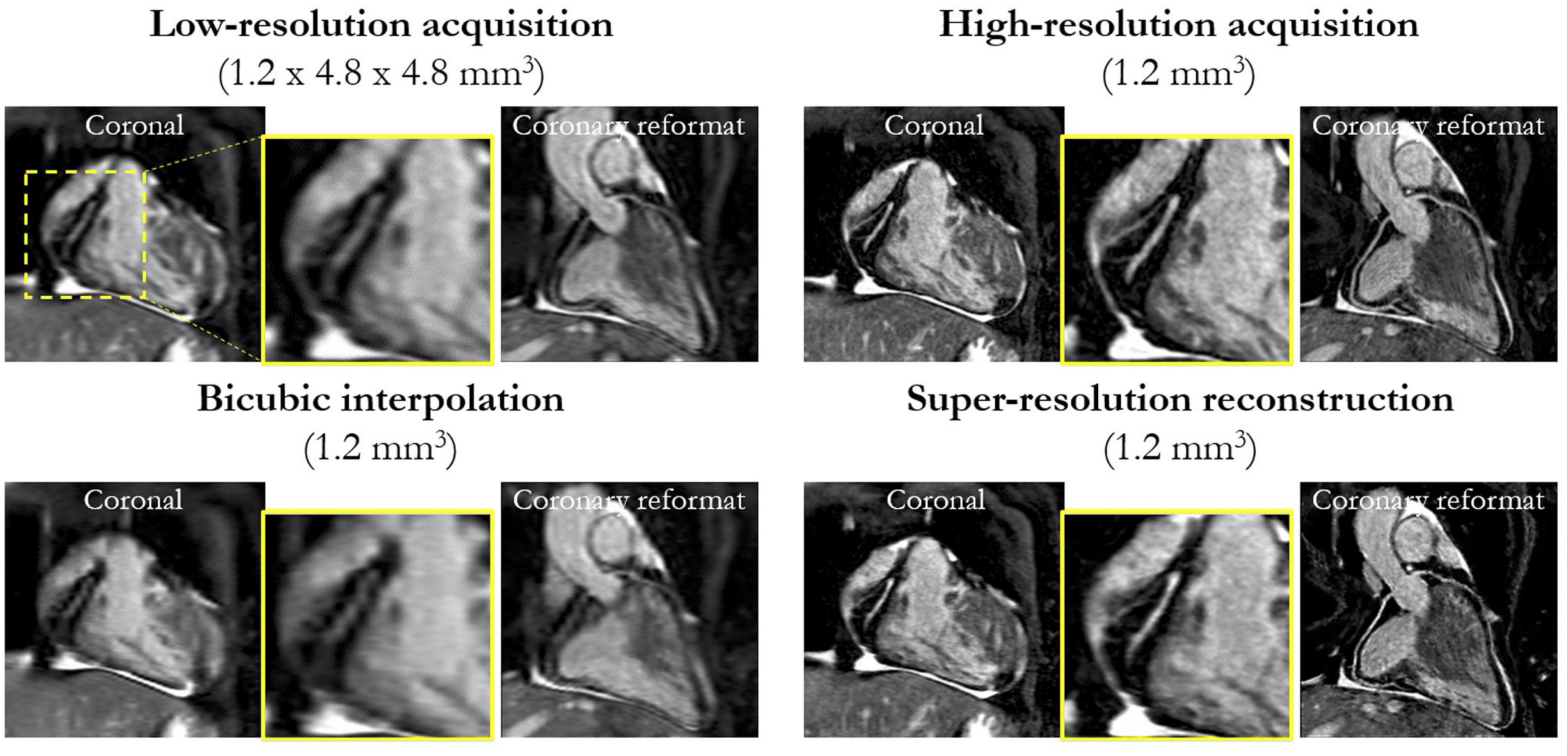
Prospective super-resolution reconstruction: coronal and coronary reformat of low-resolution acquisition (1.2 × 4.8 × 4.8 mm^3^) acquired in ~50 s compared to high-resolution acquisition (1.2 mm^3^) acquired in ~7 min. Bicubic interpolation (1.2 mm^3^) and proposed super-resolution reconstruction (1.2 mm^3^) in a patient with suspected CAD for a prospective acquired low-resolution scan (prospective cohort). Magnified image of RCA shows comparable image quality to the high-resolution acquisition in significantly shorter scan time. Küstner et al. (11). The article is published Open Access under a CC BY licence (https://creativecommons.org/licences/by/4.0/).

**Figure 4:**
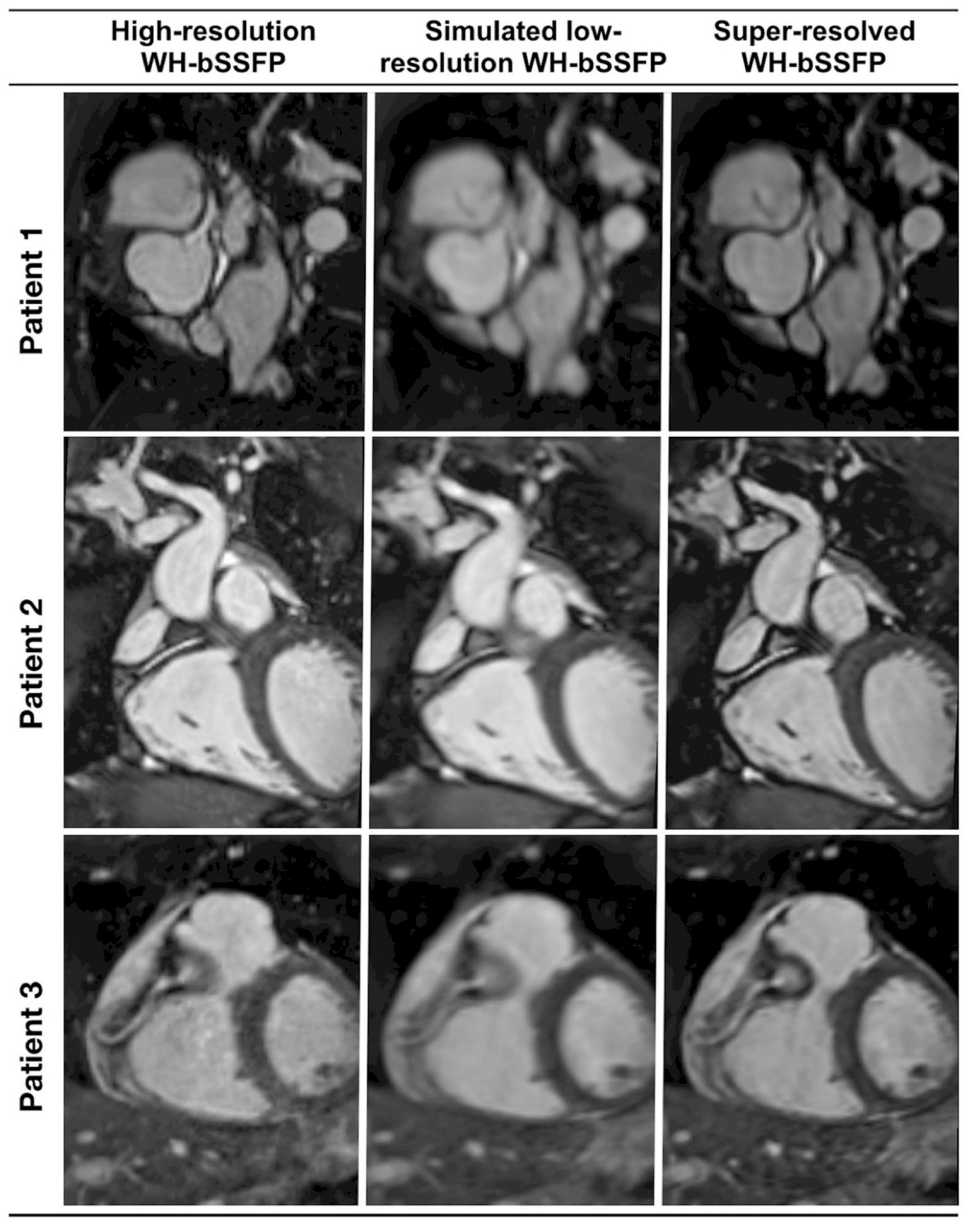
Representative image quality of the coronaries from a prospective, clinically integrated study, that utilised a residual U-Net network to facilitate super-resolution reconstruction of rapidly acquired low-resolution three-dimensional whole-heart balanced Steady State Free Precession datasets. Multi-planar reformats of the coronary artery from the respective conventional high-resolution acquisition, low-resolution acquisition, and the corresponding super-resolution reconstruction dataset. Sharpness of vascular borders is enhanced and image distortion is attenuated in the super-resolution reconstruction dataset vs. the low-resolution volume. This is particularly beneficial in the delineation of small vessels, such as the coronary arteries. Qualitative image quality analysis demonstrated no statistically significant differences between the super-resolution and the high-resolution data. Steeden et al. (13). The article is published Open Access under a CC BY licence (https://creativecommons.org/licenses/by/4.0/).

**Figure 5:**
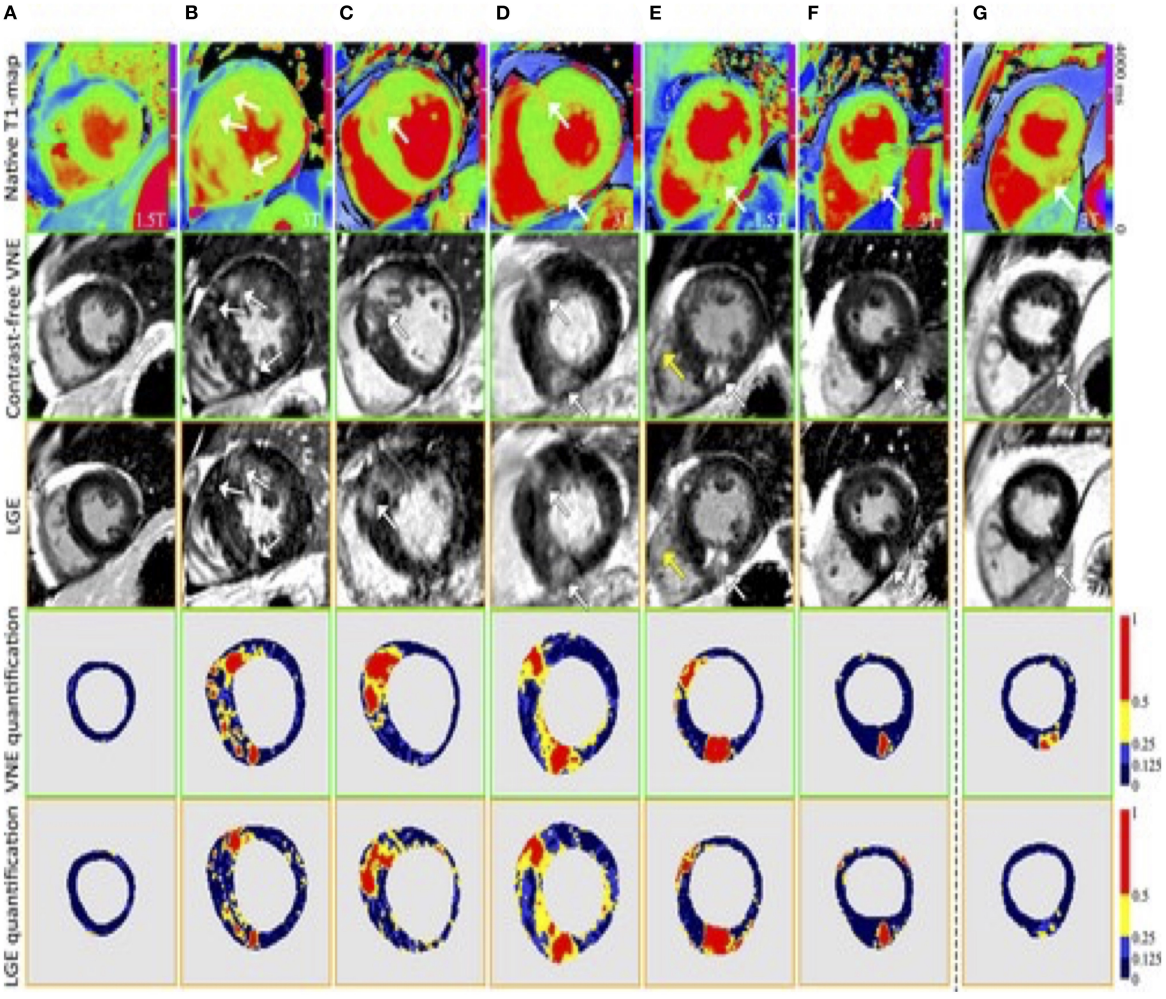
Examples to demonstrate the image quality and opticospatial correlation between VNE and conventional LGE images. T1 colormaps (top row) were adjusted to show the T1 signals that pair with the VNE signals. The bottom 2 rows visualise myocardial lesion regions by VNE and LGE using progressive thresholding (full width, at half, a quarter, and eighth maximum) displayed with different colours. In **(A–F)**, high visuospatial agreement was noted between VNE and LGE. White arrows point to the lesions. Yellow arrows point to slightly different depiction of the right ventricular wall in VNE and LGE, suggesting patient movement between acquisitions. **(G)**, An example of VNE displaying subtle changes in the distribution and quantification of the lesion clearer than LGE. LGE, late gadolinium enhancement; VNE, virtual native enhancement. Zhang et al. (16). The article is published Open Access under a CC BY licence (https://creativecommons.org/licenses/by/4.0/).

AI applications in CMR have contributed significantly to the acceleration of image acquisition and analysis. Neural networks have been applied to reconstruct data from rapidly acquired undersampled MRI images across different sequences. A deep-learning based, super-resolution CMR Angiography framework has enabled reconstruction of low resolution 1.2 x 4.8 x 4.8mm^3^ data acquired in 50 s scan time (11). The proposed method showed similar quantitative and perceivable image quality of the high resolution 1.2 mm^3^ images, achieving 16 x acceleration in acquisition time (Figure 3). Similar results have been attained with a Multi-Scale Variational Neural Network undersampled reconstruction (12), achieving 9x acceleration, in CMR Angiography 1.2 mm^3^ acquisition outperforming compressed sensing (CS) reconstruction. Steeden et al. (13) has successfully employed a subset of convolutional neural network, specifically the 3D residual U-net to perform super-resolution reconstruction on low-resolution three-dimensional whole heart balanced Steady State Free Precession (bSSFP) datasets, achieving similar diagnostic confidence and accuracy with high-resolution whole heart bSSFP in patients with CHD, Figure 4. Besides acquisition speed, AI has the potential to reduce breath-holds. Kuestner et al. (14) has introduced 9–15x acceleration in 3D cine images in a single 10–15 s breath hold utilising a DL-based approach. For a more detailed technical review of these methods, we refer the reader to a recent review by Alzubaidi et al. (15). Zhang et al. (16) developed an AI-based virtual native enhancement (VNE) imaging technology, using streams of CNN to employ and optimise the acquired signal from native T1 mapping and cine imaging sequences, depicting them as LGE-analogous images. This technology allows for contrast-free and efficient tissue characterisation, achieving high agreement in the quantification of tissue burden and superior image quality compared to the late gadolinium enhancement (LGE) images (see Figure 5) (16).

#### Segmentation

**Figure 6:**
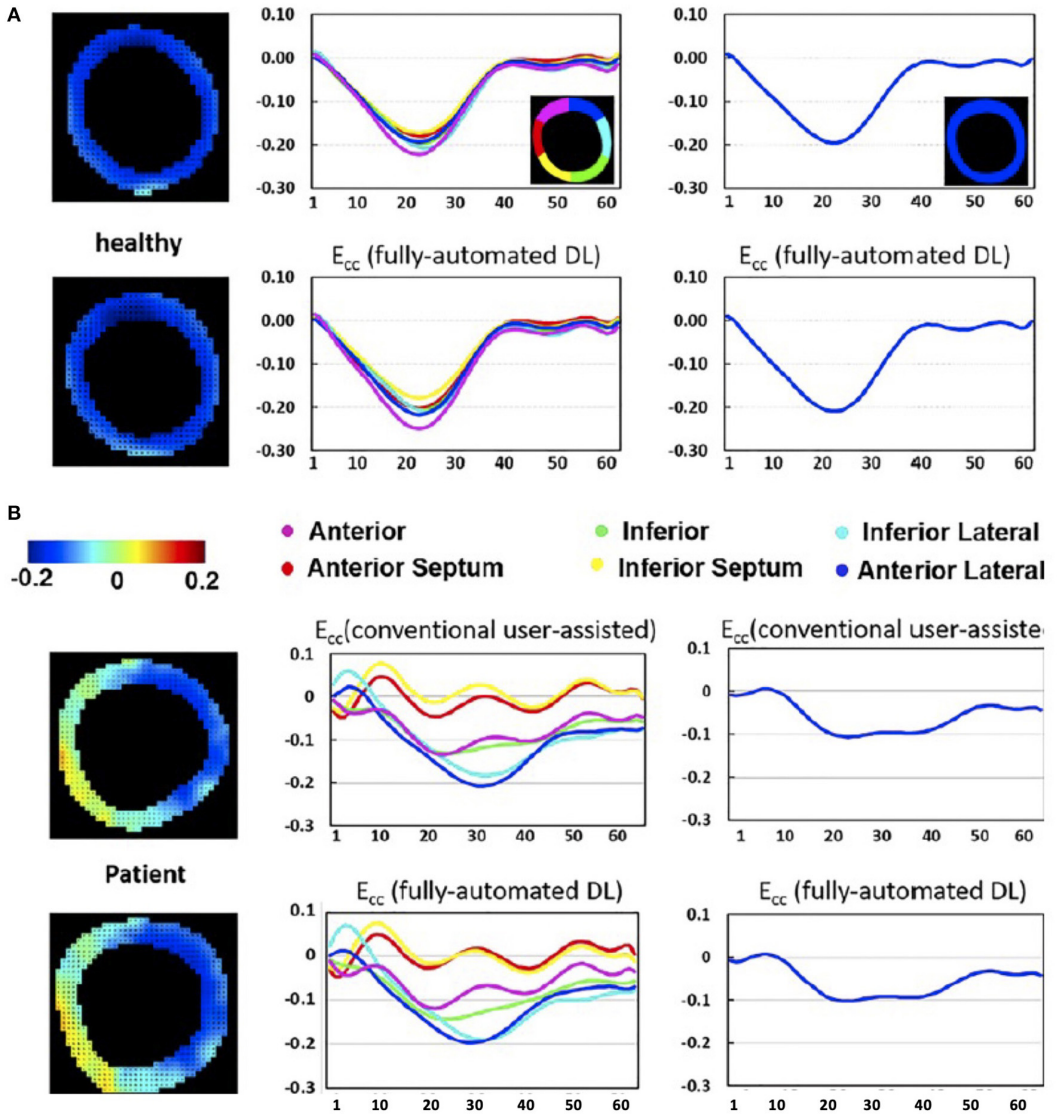
DL-based computation of global and segmental circumferential strain is compared to the clinician-assisted DENSE analysis. The AI-based end-systolic circumferential strain (Ecc) maps (left column), segmental (middle column) and global (right column) circumferential strain–time curves for a healthy subject **(A)** and a heart failure patient **(B)** demonstrate very close agreement with the conventional segmentation in the depicted mid-ventricular slices. Ghadimi et al. (26). The article is published Open Access under a CC BY licence (https://creativecommons.org/licenses/by/4.0/).

Manual delineation of image contours by experts is currently the standard clinical practise in CMR. However, this is laborious and prone to intra- and inter-observer variability. Various AI models have been proposed and clinically validated to accelerate the segmentation of right and left ventricles in adult populations (17–19). Limitations include the training in homogenous datasets like the UK Biobank (20) or cardiac atlas project, that include adult patients, the majority being with structurally normal hearts. Winther et al. (21) performed experiments utilising datasets from four independent sources for training and for validation of the network. The network proved to be capable of reliably producing high quality segmentations, independent of aspects such as different image acquisition techniques, and diverse MRI protocols and vendors. The neuronal network performed equally or outperformed the human cardiac expert in all parts of left ventricle (LV) and right ventricle (RV) volumetry and mass measurements. Bidhendi et al. (22) expanded the approach and created a fully convolutional network that was applied successfully in paediatric patients with CHD and proved to be superior to the algorithms clinically used in a commercially available platform. An extensive review on these techniques is presented in Chen et al. (23). In a recent study, employing deep fully convolutional neural network, an automated segmentation for the quantification of tissue characterisation for native T1 mapping in patients diagnosed with hypertrophic cardiomyopathy (HCM) has been developed; showing robustness in inter-observer variability and minimising analysis time to under a second (24). A similar approach was employed for automatic quantification of LV mass and scar volume on LGE images and has been successfully applied in patients post myocardial infarction (25). Additional applications of convolutional neural networks include automated phase velocity estimation and four-dimensional flow dataset segmentation along with the estimation of global and segmental myocardial strain in Displacement Encoding with stimulated echoes (DENSE) images, Figure 6 (26, 27). Significant benefits include efficient CMR reporting and high levels of reproducibility in the measurements.

### Novel Imaging Biomarkers

#### Texture Analysis/Radiomics

A recently applied technique, called texture analysis (TA), employs various ML algorithms, to quantify the spatial heterogeneity and relationship of adjacent pixels, in order to compute sophisticated imaging metrics. Texture features derived from CMR, have demonstrated potential for further research and clinical integration. It is assumed that the distribution of pixel grey-level values constitutes significant information beyond the measured mean signal. For instance, although global T1 and extra-cellular volume can differentiate HCM and Hypertensive Heart Disease from normal hearts but not between the two, as values overlap; TA features, generated though supervised ML models, have been shown to distinguish and quantitatively evaluate the subtle discrepancies between the two entities (28). A study, by Wang et al. (29) utilising similar supervised technique, is going one step further and found that TA could differentiate between patients with MYH7(b-myosin heavy chain) gene mutation from those with MYBPC3 (B-myosin binding protein C). A different approach has been investigated by other groups, who introduced motion features as a biomarker. Mancio et al. (30) exploited routine cine images from high dimensional data to objectively characterise and quantify subtle tissue alterations of the ventricular myocardium beyond the typical CMR indices in a cohort of HCM patients. The proposed method, that exploits a supervised-learning algorithm, can potentially serve as screening tool identifying HCM patients with low probability of scar, who constitute around one third of the total cohort, for whom LGE imaging would not be necessary (30). Hence, texture feature analysis could contribute to reducing patient exposure to contrast-agents and the associated service costs.

#### New Insights in Predictive Models

Various predictive models, incorporating different clinical and imaging parameters, have been introduced in cardiovascular medicine over the last decades to estimate the personalised risk for an individual patient to develop a certain outcome. A major challenge for CMR is to incorporate imaging biomarkers in clinically relevant predictive models. For the effective characterisation of cardiac disease phenotype, the use of conventional parameters of cardiac output like ejection fraction might be insufficient (31). Refined ventricular shape and motion analysis could potentially accomplish profound evaluation of cardiac motion and the extraction of its spatiotemporal patterns, which are attributed to specific diseases. Dawes et al. (32) performed computational analysis of RV 3D longitudinal, circumferential and radial motion, relative to its long-axis (defined from the tricuspid orifice and RV apex) between end-diastole and end-systole. The derived data, which represented the systolic displacement of the right ventricle and septum, were then analysed by a supervised ML algorithm, with the aim, to identify those 3D cardiac motion patterns in this high-dimensional dataset, which were more closely linked to survival. The ML survival model showed that altered contraction pattern in distinct segments of the RV free wall and septum is associated with poor prognosis and has incremental predictive performance when added to conventional biomarkers (32). In a similar direction, a fully convolutional neural network was trained to perform cardiac segmentation from hand-labelled CMR images, computing smooth time-resolved 3D renderings of the cardiac motion. Those 3D representations were employed as input data to a supervised denoising autoencoder prediction network, designed to capture robust discriminative features for survival prediction in patients with pulmonary hypertension (33). The predictive accuracy for the deep-leaning based survival model outperformed benchmark models of volumetric manually-derived CMR parameters. In a different patient group, a U-net algorithm, based on CNN architecture, was designed to automatically trace the endocardial border and calculate right atrial area and feature-tracking based strain measurements from CMR cine images in the four-chamber view and short-axis view at the papillary muscles level. Those indices, computed directly from raw medical images, correlated significantly with prognosis in a cohort of patients with repaired Tetralogy of Fallot (34). Knott et al. (35) applied a convolutional- neural network for automated quantitative myocardial perfusion analysis, in the first prospective two-centre outcome study, evaluating global mean stress myocardial blood flow and myocardial perfusion reserve with AI-based techniques. Cox hazard regression analysis demonstrated that stress myocardial blood flow and myocardial perfusion reserve were associated with events after adjusting for potential confounders and concluded that those parameters are predictive of adverse outcomes surpassing the performance of conventional cardiovascular risk factors (35). A differentiated approach was adopted by MacGregor et al. (36) who in addition to incorporating ML-derived measurements in predictive models, proposed a deep-learning based predictive clinical algorithm, advancing previously applied statistical predictive models. This preliminary investigation showed that the regional distribution patterns of machine-detected, CMR-derived, regional contractile injury could have predictive value with regards to clinical endpoints in Idiopathic Dilated Cardiomyopathy Heart Failure patients. The regional strain measurements were the input variables in a deep neural network algorithm, that could differentiate patients who responded to medical therapy from those with no response, with an area under the curve of 0.94 and 85% accuracy (36). Kotu et al. (37) incorporated CMR image-based texture features from post myocardial infarction patients, which delineate the extent, distribution, and heterogeneity of the myocardial scar in a combination of supervised ML-based algorithms and other classification methods, to distinguish between high and low arrhythmic risk group of patients.

### Current Challenges

Despite the large volume research that has been performed in CMR, real world clinical deployment of AI in clinical practise is still rare.

**Figure 7:**
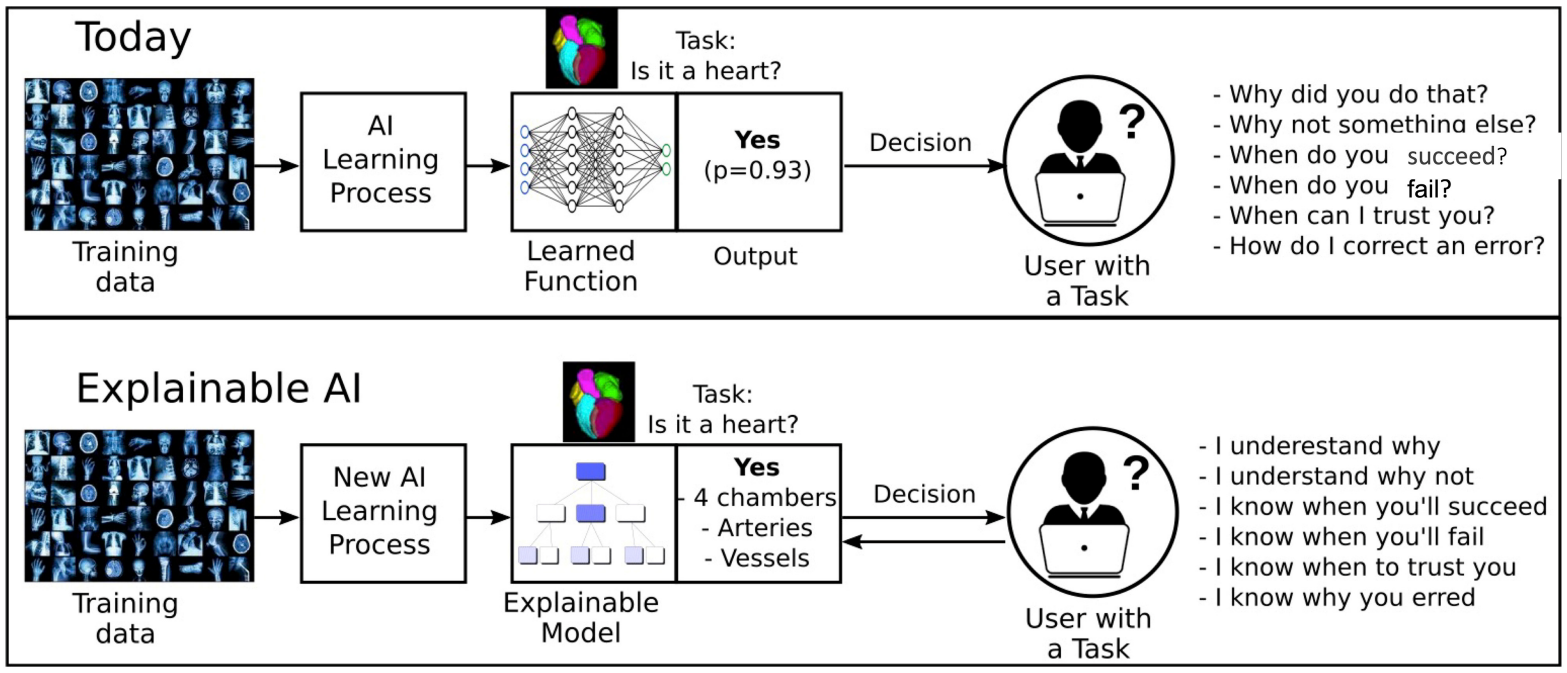
An illustrative overview of the explainable MRI concept. The user has insight in the features that influence the decision of the model.

While AI can extract novel insights from existing data, it is often difficult to justify why the network reached a certain output; the so-called “black-box problem” (38). Furthermore, regulations such as the European General Data Protection Regulation (GDPR) is enforcing the retraceability of the decision outcomes, calling into question the use of black-box models in healthcare. This calls for an approach that supports the interpretability of the machine decision-making process and the reproduction and comprehension of both the learning and knowledge extraction process. Ongoing efforts to face this challenge have resulted in the design of explainable AI models, that constitute a selection of procedures and techniques that enable human subjects to perceive and trust the outcome and the prediction derived by ML methods. In explainable AI, the expected impact and potential biases of the AI model are described. Holzinger et al. (39) presents a very helpful overview of current research topics in explainable AI. Neural network models with incorporated quality control layers are proposed. A schematic representation of the network is shown in Figure 7. Puyol-Antón et al. (40) demonstrated a novel framework to predict response to cardiac resychronisation therapy of patients with cardiomyopathy from cine cardiac imaging. The proposed model allows the extraction of visual features in the image domain of the secondary categorisation task so that the reviewer can appraise whether the learned features correlate with the clinical domain knowledge. In this method, a weakly supervised network was taught the concept of septal flash, which corresponds to a favourable response to cardiac resychronisation therapy and was able to illustrate this, by disentangling the latent space. An additional study, utilising CNNs in cardiac cine image segmentation, incorporated robust Quality Control in two distinct phases; an initial pre-analysis assessment of image quality, employing two additional CNNs, was followed by the image segmentation and computation of cardiac functional parameters. The final step was a post analysis qualitative evaluation of the output, thus allowing automated processing of considerable numbers of CMR studies, obviating the requirement for clinician's input (19).

A different approach to this problem employs the use of predictive uncertainty estimates of the segmentation model (41). The key idea is that the model generates confidence intervals of the predictions, giving insight into why the network has decided the output. Segmentation outputs with low uncertainty are likely correct while outputs with high uncertainty are likely problematic. This may improve workflow efficiency and accuracy by guiding the reviewer to focus mainly on problematic segmentations.

### Limitations Paving the Steps Forward

The greater part of AI research has utilised retrospectively acquired data. The term “AI chasm” has been introduced to express the case that the predictive accuracy of an AI model does not epitomise clinical effectiveness (42). This is because, despite the favourable results, outlining excellent network performance in preliminary, single institutional, proof-of concept studies, the adoption in clinical practise is limited and the generalisability has not been proven. Few multi-centre, multi-vendor studies have been attempted with good results that were retrospective in nature showing the feasibility of the design and encouraging the execution of similar prospective studies (43). To enhance the validity and clinical acceptance of AI applications, multi-institutional prospective studies across different clinical teams and vendors should be designed, ultimately followed by randomised controlled trials. To the best of our knowledge, in clinical CMR there is currently no prospective randomised control trial published demonstrating the clinical benefit that AI applications could potentially accomplish. Challenges that need to be faced include the lack of standardisation in image acquisition, reconstruction and analysis along with optimisation, transparency and adherence to reporting standards on trial design and methodology. Few randomised controlled trials have been conducted for different clinical applications, showing that incorporation of AI systems did not have superior outcomes when compared to the current clinical practise or decisions made the by senior clinicians (44). Lin et al. (45) showed that AI achieved high patient satisfaction due to shorter examination times, however further research is warranted as to investigate whether AI solutions can be an alternative triage tool, when a senior consultant is not available. The critical appraisal of the current studies has raised additional confounding factors influencing the methodological approaches in AI randomised control trials that could be considered for improvement in future work (46). Future directions to minimise bias would include methods to warrant effective blinding for the clinicians, inclusion of adequate number of clinicians with different levels of experience and expertise and the design of long-running studies to allow for clinicians to comprehend, adapt and utilise the AI systems effectively.

**Figure 8:**
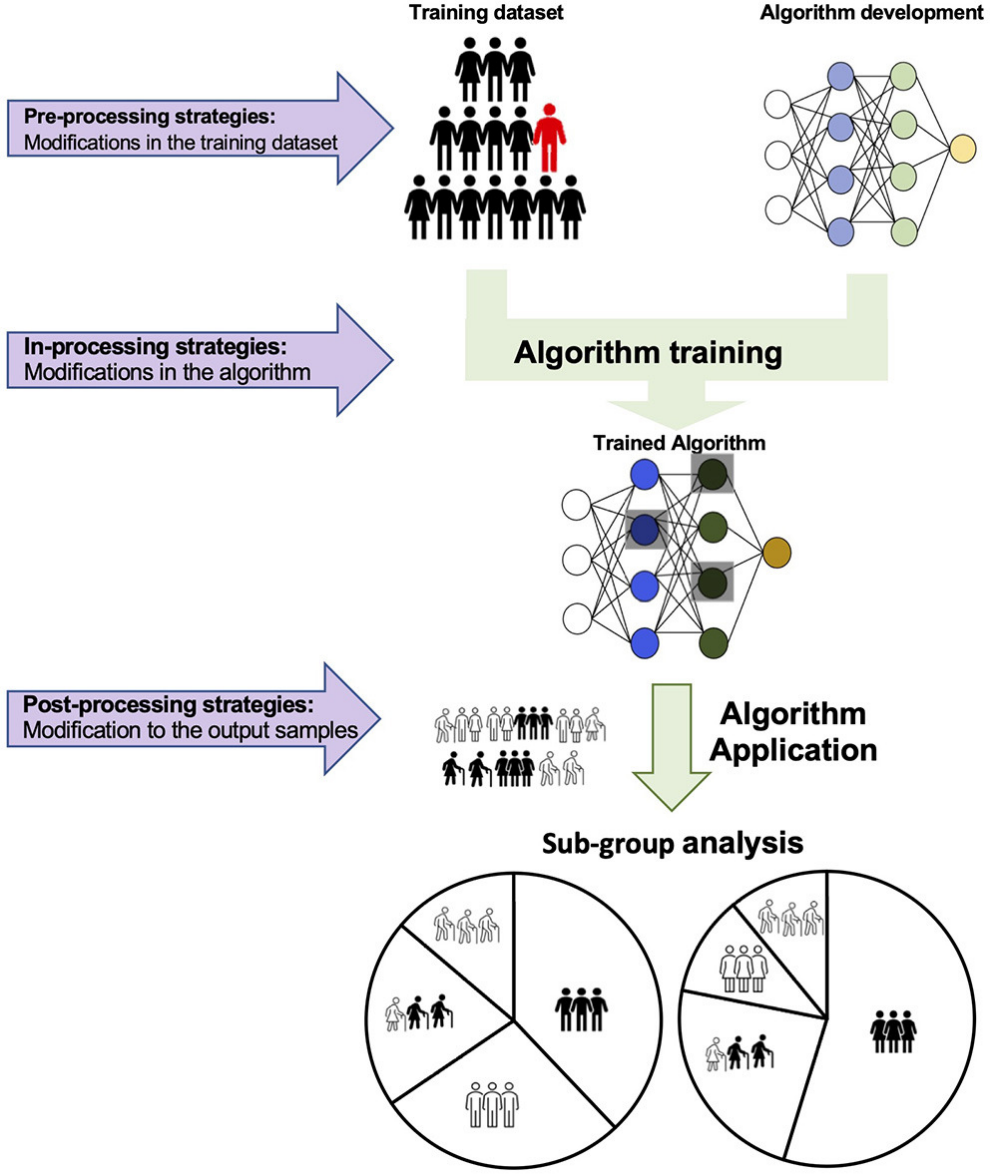
Schematic representation of three proposed strategies to introduce fairness in AI algorithms. First, pre-processing modifications in the training dataset can eliminate bias before training. In each training dataset, the data are initially classified by the protected attribute(s) (such as sex, race, ethnic origin, religious and political beliefs, age, socioeconomic background and so forth). Samples are stratified to establish equitable representation of all protected groups in the training. Alternatively, alterations in the AI algorithm can train a model to overcome discrimination and optimise the performance both in the prevalent and unprivileged group(s). The third approach attempts to train distinct models for each protected group.

Recent studies have introduced the significance of “fairness” in DL models (47), demonstrating that training data imbalance, can lead to statistically significant differences in the performance of the proposed models between different racial groups, potentially exacerbating disparities in healthcare (47). DL algorithms can be optimised to address this issue. Potential strategies to minimise bias include the modification of the training dataset to mitigate discrimination (pre-processing strategies), modifications of the learning algorithm to diminish bias (in-processing strategies) or lastly correcting the output of the applied algorithm to meet the fairness prerequisites (post-processing strategies). Figure 8 shows an example of the different bias mitigation strategies.

Meticulous clinical reporting of studies, that adopt AI methods, is critical to adequately evaluate image quality, interpret the results and assess the potential usefulness of prediction models, in order for them to be embraced in clinical routine. Forthcoming studies should include patients from diverse backgrounds and report performance per gender and race to minimise bias. It has been often stressed that the area under the curve of a receiver operating characteristic curve is not the optimal metric to assess clinical performance and is not readily comprehensible by many clinicians, although it is widely used in AI studies (48). Sensitivity and specificity should be determined at the defined model operating point (required to transform the continuous model outcome variables into discrete decision groups) and positive and negative predictive values should be reported. Published papers should include information on several measures, summarising the performance of a model, as no single measure captures all the necessary and clinically relevant properties. In addition to the extensive analysis of the results, significant attention should be paid to the practical implementation of the model and whether this achieves a favourable shift in the current patient care pathway. Hence, this is ultimately reflective of the clinical relevance of the study. For instance, various ML–based algorithms have been developed to predict hospital readmissions, showing superior predictive accuracy to conventional parameters, including initial diagnosis and demographic factors. Nevertheless, their clinical uptake is currently limited, because they fail to incorporate and measure competing parameters like clinician's time, staff availability, socio-economic background and so on (49). To progress the comprehension and clinical integration of ML research studies, researchers are asked to adhere to best practise guidance, such as the Transparent Reporting of a multivariable prediction model for Individual Prognosis Or Diagnosis (TRIPOD), developed to support the thorough and transparent reporting of studies that design, validate or update a prediction model (50). An additional version of the TRIPOD statement that is tailored to ML prediction algorithms (TRIPOD-ML) is in progress. This will be intended for the development of a robust framework to provide methodological and reporting guidance for ML studies in healthcare (51).

**Figure 9:**
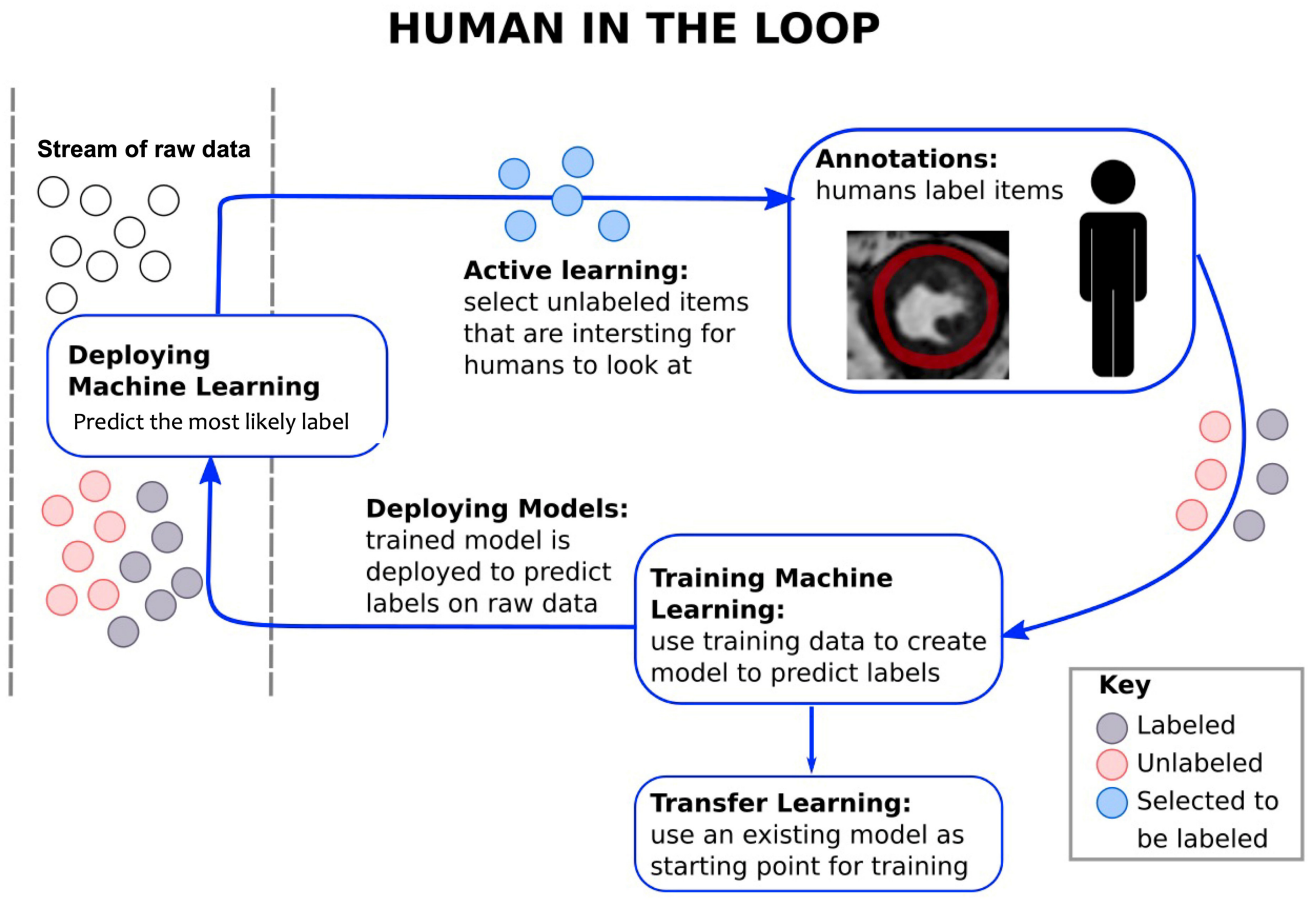
Brief chart on the framework of “clinician in the loop.” Clinicians are provided with action choices. Data labelled from clinicians contribute to the training of the network.

A recently introduced concept, that is promising and, as far as we know, has not yet been adopted in cardiac MRI is the so-called clinician in-the-loop (52). This is a type of reinforcement learning, where the model keeps learning based on the input of the clinician (Figure 9). Further studies are required to investigate whether this method can improve the quality of AI applications in different tasks, including segmentation and development of predictive models along with gaining clinicians' trust.

### Clinician Engagement

At present, clinician's input is mandatory not only in labelling the data and appraising the developed frameworks, but more importantly in the decision-making process. Most clinicians are currently far away from entrusting computers to match the comprehensive skills of a radiologist. While it is important to encourage the adoption of an AI curriculum for medical students and practising clinicians to allow them to critically review, evaluate and apply AI tools safely in clinical routine, excessive confidence in AI technology is not yet topical. Clinical skills, starting from elaborate history taking, to physical examination along with the enriching and therapeutic patient-physician relationship have been the mainstay of medicine for centuries and should constantly be fostered and harnessed in the parallel development and application of AI tools.

In the years to come, a dedicated collaboration between computer scientists, medical imaging physicists and clinicians in CMR is promising exciting strides in this field. Explainable AI techniques are expected to enable faster integration of AI models into the clinical practise, and will aid in fostering the necessary integrity and trust with their users.

## Conclusion

AI is envisaged as a useful tool to accelerate CMR imaging acquisition, analysis and reporting, while introducing new diagnostic and prognostic biomarkers. Careful design and assessment of future studies alongside improved interpretability of the algorithms and enhanced clinician's input will accelerate potential clinical adoption.

## Author Contributions

AF, EP-A, KP, and CP devised and wrote the manuscript. AC and RB reviewed the manuscript. All authors contributed to the article and approved the submitted version.

## Funding

The authors acknowledge financial support from the BHF PG/18/59/33955, EPSRC EP/P001009, EP/P032311/1, EP/P007619, Wellcome EPSRC Centre for Medical Engineering (NS/A000049/1), and the Department of health via the National Institute for Health Research (NIHR) comprehensive Biomedical Research Centre award to Guy's and St. Thomas' NHS Foundation Trust. The views expressed are those of the authors and not necessarily those of the NHS, the NIHR, or the Department of Health. This research was funded in part, by the Wellcome Trust (NS/A000049/1). For the purpose of open access, the author has applied a CC BY public copyright licence to any Author Accepted Manuscript version arising from this submission.

## Conflict of Interest

The authors declare that the research was conducted in the absence of any commercial or financial relationships that could be construed as a potential conflict of interest.

## Publisher's Note

All claims expressed in this article are solely those of the authors and do not necessarily represent those of their affiliated organizations, or those of the publisher, the editors and the reviewers. Any product that may be evaluated in this article, or claim that may be made by its manufacturer, is not guaranteed or endorsed by the publisher.

## Abbreviations

AI: Artificial Intelligence
CHD: Congenital Heart Disease
CMR: Cardiovascular Magnetic Resonance
CNN: Convolutional Neural Network
DL: Deep-learning
HCM: Hypertrophic Cardiomyopathy
LGE: Late-gadolinium enhancement
LV: Left ventricle
ML: Machine-learning
MRI: Magnetic Resonance Imaging
RV: Right ventricle
TA: Texture analysis
TRIPOD: Transparent Reporting of a multivariable prediction model for Individual Prognosis Or Diagnosis
3D: Three-dimensional
4D: Four-dimensional.

## References

[1] Hao Z Shyam RBA Rathinam A Gao Y. Intelligent spacecraft visual GNC architecture with the state-of-the-art AI components for on-orbit manipulation. Front Robot AI. (2021) 8:639327. 10.3389/frobt.2021.639327 34141728 PMC8204185

[2] Huang B Ji Z Zhai R Xiao C Yang F Yang B. Clock bias prediction algorithm for navigation satellites based on a supervised learning long short-term memory neural network. GPS Solutions. (2021) 25:80. 10.1007/s10291-021-01115-0

[3] Ding M. A second generation of the neural network model for predicting weighted mean temperature. GPS Solutions. (2020) 24:1–6. 10.1007/s10291-020-0975-3

[4] Erickson BJ Korfiatis P Akkus Z Kline TL. Machine learning for medical imaging. Radiographics. (2017) 37:505–15. 10.1148/rg.2017160130 28212054 PMC5375621

[5] Sony S Dunphy K Sadhu A Capretz M. A systematic review of convolutional neural network-based structural condition assessment techniques. Engineering Structures. (2021) 226:11137. 10.1016/j.engstruct.2020.111347

[6] Ravi D Wong C Deligianni F Berthelot M Andreu-Perez J Lo B. Deep Learning for Health Informatics. IEEE J Biomed Health Inform. (2017) 21:4–21. 10.1109/JBHI.2016.2636665 28055930

[7] Keenan NG Captur G McCann GP Berry C Myerson SG Fairbairn T. Regional variation in cardiovascular magnetic resonance service delivery across the UK. Heart. (2021). 10.1136/heartjnl-2020-318667 33766986 PMC8639953

[8] Scannell CM Veta M Villa ADM Sammut EC Lee J Breeuwer M. Deep-learning-based preprocessing for quantitative myocardial perfusion MRI. J Magn Reson Imaging. (2020) 51:1689–96. 10.1002/jmri.26983 31710769 PMC7317373

[9] Menacho K Ramirez S Segura P Nordin S Abdel-Gadir A Illatopa V. INCA (Peru) study: impact of non-invasive cardiac magnetic resonance assessment in the developing world. J Am Heart Assoc. (2018) 7:e008981. 10.1161/JAHA.118.008981 30371164 PMC6201420

[10] Leiner T Rueckert D Suinesiaputra A Baessler B Nezafat R Isgum I. Machine learning in cardiovascular magnetic resonance: basic concepts and applications. J Cardiovasc Magn Reson. (2019) 21:61. 10.1186/s12968-019-0575-y 31590664 PMC6778980

[11] Kustner T Munoz C Psenicny A Bustin A Fuin N Qi H. Deep-learning based super-resolution for 3D isotropic coronary MR angiography in less than a minute. Magn Reson Med. (2021) 86:2837–52. 10.1002/mrm.28911 34240753

[12] Fuin N Bustin A Kustner T Oksuz I Clough J King AP. A multi-scale variational neural network for accelerating motion-compensated whole-heart 3D coronary MR angiography. Magn Reson Imaging. (2020) 70:155–67. 10.1016/j.mri.2020.04.007 32353528

[13] Steeden JA Quail M Gotschy A Mortensen KH Hauptmann A Arridge S. Rapid whole-heart CMR with single volume super-resolution. J Cardiovasc Magn Reson. (2020) 22:56. 10.1186/s12968-020-00651-x 32753047 PMC7405461

[14] Kustner T Fuin N Hammernik K Bustin A Qi H Hajhosseiny R. CINENet: deep learning-based 3D cardiac CINE MRI reconstruction with multi-coil complex-valued 4D spatio-temporal convolutions. Sci Rep. (2020) 10:13710. 10.1038/s41598-020-70551-8 32792507 PMC7426830

[15] Alzubaidi L Zhang J Humaidi AJ Al-Dujaili A Duan Y Al-Shamma O. Review of deep learning: concepts, CNN architectures, challenges, applications, future directions. J Big Data. (2021) 8:53. 10.1186/s40537-021-00444-8 33816053 PMC8010506

[16] Zhang Q Burrage MK Lukaschuk E Shanmuganathan M Popescu IA Nikolaidou C. Toward replacing late gadolinium enhancement with artificial intelligence virtual native enhancement for gadolinium-free cardiovascular magnetic resonance tissue characterization in hypertrophic cardiomyopathy. Circulation. (2021) 144:589–99. 10.1161/circulationaha.121.054432 34229451 PMC8378544

[17] Duan J Bello G Schlemper J Bai W Dawes TJW Biffi C. Automatic 3D Bi-ventricular segmentation of cardiac images by a shape-refined multi- task deep learning approach. IEEE Trans Med Imaging. (2019) 38:2151–64. 10.1109/TMI.2019.2894322 30676949 PMC6728160

[18] Avendi MR Kheradvar A Jafarkhani H. A combined deep-learning and deformable-model approach to fully automatic segmentation of the left ventricle in cardiac MRI. Med Image Anal. (2016) 30:108–19. 10.1016/j.media.2016.01.005 26917105

[19] Ruijsink B Puyol-Anton E Oksuz I Sinclair M Bai W Schnabel JA. Fully automated, quality-controlled cardiac analysis from CMR: validation and large-scale application to characterize cardiac function. JACC Cardiovasc Imaging. (2020) 13:684–95. 10.1016/j.jcmg.2019.05.030 31326477 PMC7060799

[20] Sudlow C Gallacher J Allen N Beral V Burton P Danesh J. UK Biobank: an open access resource for identifying the causes of a wide range of complex diseases of middle and old age. PLoS Med. (2015) 12:e1001779. 10.1371/journal.pmed.1001779 25826379 PMC4380465

[21] Winther HB Hundt C Schmidt B Czerner C Bauersachs J Wacker F. ν-net: deep learning for generalized biventricular mass and function parameters using multicenter cardiac MRI data. JACC Cardiovasc Imaging. (2018) 11:1036–8. 10.1016/j.jcmg.2017.11.013 29361481

[22] Karimi-Bidhendi S Arafati A Cheng AL Wu Y Kheradvar A Jafarkhani H. Fullyautomated deeplearning segmentation of pediatric cardiovascular magnetic resonance of patients with complex congenital heart diseases. J Cardiovasc Magn Reson. (2020) 22:80. 10.1186/s12968-020-00678-0 33256762 PMC7706241

[23] Chen C Qin C Qiu H Tarroni G Duan J Bai W. Deep learning for cardiac image segmentation: a review. Front Cardiovasc Med. (2020) 7:25. 10.3389/fcvm.2020.00025 32195270 PMC7066212

[24] Fahmy AS El-Rewaidy H Nezafat M Nakamori S Nezafat R. Automated analysis of cardiovascular magnetic resonance myocardial native T1 mapping images using fully convolutional neural networks. J Cardiovasc Magn Reson. (2019) 21:7. 10.1186/s12968-018-0516-1 30636630 PMC6330747

[25] Fahmy AS Rausch J Neisius U Chan RH Maron MS Appelbaum E. Automated cardiac MR scar quantification in hypertrophic cardiomyopathy using deep convolutional neural networks. JACC Cardiovasc Imaging. (2018) 11:1917–8. 10.1016/j.jcmg.2018.04.030 30121270 PMC6286195

[26] Ghadimi S Auger DA Feng X Sun C Meyer CH Bilchick KC. Fully-automated global and segmental strain analysis of DENSE cardiovascular magnetic resonance using deep learning for segmentation and phase unwrapping. J Cardiovasc Magn Reson. (2021) 23:20. 10.1186/s12968-021-00712-9 33691739 PMC7949250

[27] Bratt A Kim J Pollie M Beecy AN Tehrani NH Codella N. Machine learning derived segmentation of phase velocity encoded cardiovascular magnetic resonance for fully automated aortic flow quantification. J Cardiovasc Magn Reson. (2019) 21:1. 10.1186/s12968-018-0509-0 30612574 PMC6322266

[28] Neisius U El-Rewaidy H Nakamori S Rodriguez J Manning WJ Nezafat R. Radiomic analysis of myocardial native T1 imaging discriminates between hypertensive heart disease and hypertrophic cardiomyopathy. JACC Cardiovasc Imaging. (2019) 12:1946–54. 10.1016/j.jcmg.2018.11.024 30660549 PMC7032053

[29] Wang J Yang F Liu W Sun J Han Y Li D. Radiomic analysis of native T1 mapping images discriminates between MYH7 and MYBPC3-related hypertrophic cardiomyopathy. J Magn Reson Imaging. (2020) 52:1714–21. 10.1002/jmri.27209 32525266

[30] Mancio J Pashakhanloo F El-Rewaidy H Jang J Joshi G Csecs I. Machine learning phenotyping of scarred myocardium from cine in hypertrophic cardiomyopathy. Eur Heart J Cardiovasc Imaging. (2021) jeab056. 10.1093/ehjci/jeab056. [Online ahead of print]. 33779725 PMC9125682

[31] Sinclair M Peressutti D Puyol-Anton E Bai W Rivolo S Webb J. Myocardial strain computed at multiple spatial scales from tagged magnetic resonance imaging: Estimating cardiac biomarkers for CRT patients. Med Image Anal. (2018) 43:169–85. 10.1016/j.media.2017.10.004 29112879

[32] Dawes TJW de Marvao A Shi W Fletcher T Watson GMJ Wharton J. Machine learning of three-dimensional right ventricular motion enables outcome prediction in pulmonary hypertension: a cardiac MR imaging study. Radiology. (2017) 283:381–90. 10.1148/radiol.2016161315 28092203 PMC5398374

[33] Bello GA Dawes TJW Duan J Biffi C de Marvao A Howard LSGE. Deep-learning cardiac motion analysis for human survival prediction. Nat Mach Intell. (2019) 1:95–104. 10.1038/s42256-019-0019-2 30801055 PMC6382062

[34] Diller GP Orwat S Vahle J Bauer UMM Urban A Sarikouch S. Prediction of prognosis in patients with tetralogy of Fallot based on deep learning imaging analysis. Heart. (2020) 106:1007–14. 10.1136/heartjnl-2019-315962 32161041

[35] Knott KD Seraphim A Augusto JB Xue H Chacko L Aung N. The prognostic significance of quantitative myocardial perfusion: an artificial intelligence-based approach using perfusion mapping. Circulation. (2020) 141:1282–91. 10.1161/CIRCULATIONAHA.119.044666 32078380 PMC7176346

[36] MacGregor RM Guo A Masood MF Cupps BP Ewald GA Pasque MK. Machine learning outcome prediction in dilated cardiomyopathy using regional left ventricular multiparametric strain. Ann Biomed Eng. (2021) 49:922–32. 10.1007/s10439-020-02639-1 33006006 PMC7854526

[37] Kotu LP Engan K Borhani R Katsaggelos AK Orn S Woie L. Cardiac magnetic resonance image-based classification of the risk of arrhythmias in post-myocardial infarction patients. Artif Intell Med. (2015) 64:205–15. 10.1016/j.artmed.2015.06.001 26239472

[38] Singh A Sengupta S Lakshminarayanan V. Explainable deep learning models in medical image analysis. J Imaging. (2020) 6:52. 10.3390/jimaging6060052 34460598 PMC8321083

[39] Holzinger A Biemann C Pattichis C Kell D. What do we need to build explainable AI systems for the medical domain? *arXiv preprint arXiv:1712.09923*. (2017).

[40] Puyol-Anton E Chen C Clough JR Ruijsink B Sidhu BS Gould J. Interpretable deep models for cardiac resynchronisation therapy response prediction. Med Image Comput Comput Assist Interv. (2020) 2020:284–93. 10.1007/978-3-030-59710-8_28 34109325 PMC7610934

[41] Ng M Guo F Biswas L Petersen SE Piechnik SK Neubauer S. Estimating Uncertainty in Neural Networks for Cardiac MRI Segmentation: A Benchmark Study 2020 Available online at: https://ui.adsabs.harvard.edu/abs/2020arXiv201215772N (accessed December 01, 2020). 10.1109/TBME.2022.3232730 37015623

[42] Keane PA Topol EJ. With an eye to AI and autonomous diagnosis. NPJ Digit Med. (2018) 1:40. 10.1038/s41746-018-0048-y 31304321 PMC6550235

[43] Tao Q Yan W Wang Y Paiman EHM Shamonin DP Garg P. Deep learning-based method for fully automatic quantification of left ventricle function from cine MR images: a multivendor, multicenter study. Radiology. (2019) 290:81–8. 10.1148/radiol.2018180513 30299231

[44] Brocklehurst P Field D Greene K Juszczak E Keith R Kenyon S. Computerised interpretation of fetal heart rate during labour (INFANT): a randomised controlled trial. Lancet. (2017) 389:1719–29. 10.1016/S0140-6736(17)30568-8 28341515 PMC5413601

[45] Lin H Li R Liu Z Chen J Yang Y Chen H. Diagnostic efficacy and therapeutic decision-making capacity of an artificial intelligence platform for childhood cataracts in eye clinics: a multicentre randomized controlled trial. EClinicalMedicine. (2019) 9:52–9. 10.1016/j.eclinm.2019.03.001 31143882 PMC6510889

[46] Genin K Grote T. Randomized controlled trials in medical AI a methodological critique. Philos Med. (2021) 2. 10.5195/philmed.2021.27

[47] Puyol-Antón E Ruijsink B Harana JM Piechnik SK Neubauer S Petersen SE. Fairness in cardiac magnetic resonance imaging: assessing sex and racial bias in deep learning-based segmentation. medRxiv. (2021). 10.1101/2021.07.19.21260749 PMC9021445 35463778

[48] Saito T Rehmsmeier M. The precision-recall plot is more informative than the ROC plot when evaluating binary classifiers on imbalanced datasets. PLoS ONE. (2015) 10:e0118432. 10.1371/journal.pone.0118432 25738806 PMC4349800

[49] Shah NH Milstein A Bagley DS. Making machine learning models clinically useful. JAMA. (2019) 322:1351–2. 10.1001/jama.2019.10306 31393527

[50] Collins GS Reitsma JB Altman DG Moons KG. Transparent reporting of a multivariable prediction model for individual prognosis or diagnosis (TRIPOD): the TRIPOD statement. BMJ. (2015) 350:g7594. 10.1136/bmj.g7594 25569120

[51] Collins GS Dhiman P Andaur Navarro CL Ma J Hooft L Reitsma JB. Protocol for development of a reporting guideline (TRIPOD-AI) and risk of bias tool (PROBAST-AI) for diagnostic and prognostic prediction model studies based on artificial intelligence. BMJ Open. (2021) 11:e048008. 10.1136/bmjopen-2020-048008 34244270 PMC8273461

[52] Tang S Modi A Sjoding M Wiens J. Clinician-in-the-loop decision making: reinforcement learning with near-optimal set-valued policies. In: Hal D, Aarti S, editors. Proceedings of the 37th International Conference on Machine Learning; Proceedings of Machine Learning Research. (2020). p. 9387–96.

